# A Narrative Case Study of Chinese Senior High School English Teachers’ Emotions: An Ecological Perspective

**DOI:** 10.3389/fpsyg.2021.792400

**Published:** 2021-12-10

**Authors:** Xiaohui Sun, Liu Yang

**Affiliations:** School of Foreign Languages and Literature, Beijing Normal University, Beijing, China

**Keywords:** teacher emotion, narrative case study, ecological system framework, ecological factors, teachers’ professional development

## Abstract

Teacher emotion research is of great significance to teachers’ teaching effectiveness, professional development, and physical and mental health. Taken from an ecological perspective, this narrative case study used purposeful sampling to select two Chinese senior high school English teachers as research participants. Various data collection methods were used, including narrative framework, teacher interview and teacher reflection log, to describe the emotional episodes of Chinese senior high school English teachers before and after collective lesson presentation, trial teaching, and formal teaching in a teaching improvement project. The purpose of this collection of data was to explore the dynamic emotional development process and characteristics of Chinese senior high school English teachers in the interaction with ecological systems and those ecological factors that may influence their emotional development. Results indicated that the two participants developed 68 emotions: 39 positive and 29 negative emotions. At exosystem, they developed the most emotions (28 emotions). Teacher emotion changed with time quite obviously. They evolved from positive to negative and, finally, predominantly positive. Personal antecedents, contextual antecedents, and teachers’ emotional capacity are the main ecological factors that may influence the development of teacher emotion. Based on the research findings, implications for teachers’ professional development and teacher education were also provided.

## Introduction

Emotions are one of the core parts of human life and “are an integral part of education and of organizations more generally” (Hargreaves, 2000, p. 812). As Schutz and Lanehart (2002) have argued, “emotions are intimately involved in virtually every aspect of the teaching and learning process” (p. 67). Teachers are emotional beings (Zembylas, 2005). They constantly experience emotional demands from students, colleagues, parents, and leaders in emotional arenas such as schools and classrooms (Cross and Hong, 2012). Since the late 1990s, research on teacher emotion in education has received more and more attention. Studies have revealed that teacher emotion affects all aspects of their professional development, such as teachers’ behavior, teaching, personal lives, professional identity, educational reform, and students’ learning (Hargreaves, 2005; Cheng, 2006; Li et al., 2013; Hagenauer and Volet, 2014; Schutz, 2014). Since the beginning of this century, with the rise of humanism in the world, teacher emotion research has gradually attracted more and more attention, and has shown rapid development since 2008 (Hu and Wang, 2014). Through ongoing research, the perspective on teacher emotion has undergone several changes. It has shifted from the psychological perspective to the sociological perspective, and, more recently, to the post-structuralist and ecological perspectives (Gu and Gu, 2015). Teacher emotion is increasingly regarded as person-environment transactions rather than just teachers’ internal personal feelings (Schutz et al., 2006). In addition, more attention is being drawn to the “link between microscopic perspectives focused at the level of ‘teacher self’ and the macroscopic level of social, cultural and political structures of schooling” (Zembylas, 2011, p. 31). This ecological perspective takes a comprehensive and systematic approach to understanding and exploring teacher emotion and teachers’ external experiences based on emotional episodes (Gu and Gu, 2015). In recent years, more and more researchers have adopted this new perspective to study teacher emotion, such as Cross and Hong (2012) and Chen (2017, 2019). However, relative studies are not in full flourish. That is, more studies investigating teacher emotion from ecological perspective are needed, especially on foreign language teacher emotion. What is more, current studies on teacher emotion from ecological perspective have shown several drawbacks: firstly, the research subject is relatively too focused—secondary school foreign language teachers receive less attention; secondly, interview seems to be one and only data collection method in most studies, influencing the richness of the results; and, finally, as for the research questions to be probed into, the investigations on the influential factors leading to the development of teacher emotion are neglected to a large extent.

Therefore, based on above research gaps, this study primarily aims at investigating secondary school foreign language teacher emotion both internally and externally by means of using multiple data collection methods. To be more concrete, we hope to gain insight into how the internal personal characteristics and external environments interact to constitute their emotions. A second aim is to probe into the possible ecological factors that influence the development of teacher emotion in different ecological systems. In accordance with the two aims, two research questions are addressed: (1) How did Chinese senior high school English teachers develop their emotions in the interactions with different ecological systems during their participation in a teaching improvement project? (2) What are the ecological factors that may influence Chinese senior high school English teachers’ emotional development? To better answer the two questions, this study focuses on two senior high school English teachers in Beijing, China, who are participating in a teaching improvement project. Throughout this project, teachers interacted with experts who provided suggestions about improvement and also cooperated with their colleagues for help. In their interactions with different people and experiences of different matters from various systems, their emotions showed great changes. By conducting this narrative case study on two teachers during their teaching improvement project, the study offers potential contributions to the literature by innovating research methods that adopt multiple data collection methods to gain rich data of teacher emotion. Especially through the participants’ self-reported narration of their emotion development, the authenticity and richness of the data are guaranteed, based on which deep qualitative analysis of teacher emotion can be conducted. The study will also contribute to expand research content of studies on teacher emotion by not only examining the development of teacher emotion but also probing into the reasons for specific emotions. As for practice of teacher emotion, this study provides implications for teachers’ professional development and teacher education.

## Literature Review

### Defining Teacher Emotion

Sutton and Wheatley (2003) suggest that teacher emotion includes appraisal, subjective experience, physiological changes and emotional expression, and action tendencies. However, this definition mainly reflects a psychological perspective without considering the contributions of sociocultural and environmental factors to an individual teacher emotion. Schutz et al. (2006) defined emotion as “socially constructed, personally enacted ways of beings that emerge from conscious and/or unconscious judgments regarding perceived successes at attaining goals or maintaining standards or beliefs during transactions as part of social-historical contexts” (p. 344). This definition rests on the assumption that teacher emotion is related to the environment in a particular context. According to Zembylas (2005), the various cultural, social, and even political factors have significant effects on how, why, and when people develop, manage, and show emotions during the transaction with the environment. Thus, teacher emotion does not exist within an individual or environment independently; rather, it involves person-environment transactions (Schutz et al., 2006). This perspective on teacher emotion reflects teachers’ sensations of interacting with students, peers, parents, and others in a particular environment rather than being generated internally (Farouk, 2012), is consistent with an ecological perspective, and, therefore, is the definition that will be used in this study.

### Classification of Emotions

Emotions have been classified into several categories, including dichotomous, multiple, and dimensional. The dichotomous classification categorizes teacher emotion into positive and negative. Positive emotions include joy, satisfaction, pride, and excitement; negative emotions include anger, frustration, anxiety, and sadness (Hargreaves, 1998). This classification is common but is also claimed to be too straightforward (Sutton and Wheatley, 2003). Multiple and dimensional categories have also been criticized for not acknowledging a reasonable quantity of basic emotions (Ortony and Turner, 1990). Parrott (2001) described a more comprehensive list of emotions that organizes emotions into a short tree structure where basic emotions are divided into secondary emotions, which are, in turn, subdivided into tertiary ones. The first level includes six basic emotions: love, joy, surprise, anger, sadness, and fear. The first level is followed by more secondary emotions. Joy, for example, is followed by cheerfulness, zest, contentment, pride, optimism, enthrallment, and relief. And each secondary emotion group has tertiary divisions. Parrott’s classification details a vast list of specific and superficial tertiary emotions and of deeper secondary and primary emotions (Chen, 2016). Therefore, this study will categorize teacher emotion based on this tree structure because it “gives a full account of human emotions and provides insightful awareness of the way emotions are linked to deeper categories” (p. 70). Finally, these emotions will be categorized into positive and negative ones.

### Studies of Teacher Emotion in the Ecological Framework

Teacher emotion as the sub-theme of psychology of teachers is one of the three major themes for studying foreign language teachers’ development from ecological perspective (Liu, 2021). In order to correspond to the two research questions in this study, the relevant research will be reviewed from two aspects: the development of teacher emotion and the influential factors leading to different emotions. The research contents and research methods are as follows:

Most of the studies on the development of teacher emotion according to the ecological framework used qualitative research design such as case study and narrative inquiry. Cross and Hong (2012) conducted a qualitative case study, discussing two elementary teachers’ emotions during their services for a high-poverty and high-minority population in the United States. The study reported that the teachers experienced different positive and negative emotions in different systems and generated a person-environmental interaction model. Chen (2017) explored 53 primary teachers’ emotional experience in their teaching journeys in Hong Kong and mainland China. These teachers described the same amount of positive and negative emotions. And different types of emotions decrease as distance from the teachers increases the five nested ecological systems. That is, teachers experienced fewer emotions with and within the environments further from them. Then, in 2019, Chen innovated the research method and employed a mixed method to further examine 1,492 primary teachers’ emotions in China. Evidence from both the qualitative and quantitative data demonstrated that a high intensity of emotions is presented at the microsystem level. In addition, this study found that the number of emotions that teachers reported decreased as the distance between the teachers and each system increased. Simonton et al. (2021) took PE teachers as participants and used ecological dynamic systems theory to position their emotions within teachers’ classrooms and their sociopolitical and cultural experiences. This study proposed a conceptual framework for understanding teacher emotion that accounts for the dynamic, evolving, and complex contexts in which teachers work, but it is not specifically for language teacher emotion.

As for the studies on ecological factors leading to the development of teacher emotion, the researchers tried to investigate and summarize the factors from different perspectives, focusing on teachers’ interaction with different systems. Berg and Cornell (2016) considered school climate as the main factor and examined whether schools with high disciplinary structure and student support were associated with less teacher aggression and distress, and found that more structured and supportive schools relate to greater safety for teachers and, in turn, less distress. Schmidt et al. (2017) focused on the day-to-day experience of beginning teachers’ first years in school and found that teaching classes and their interaction with colleagues are the main factors that lead to their daily uplifts and hassles. To better analyze and induct various influential factors, several models or frameworks were built. Richards et al. (2018) presented a model for understanding how the school environment influences teacher burnout. This model highlights the importance of developing optimal working conditions that nurture teacher development. However, it only focuses on one aspect of teacher emotion–burnout, ignoring teachers’ positive emotional development. Cross and Hong’s (2012) person-environmental interaction model explains the personal and ecological factors that influence the formation of teacher emotion. However, it does not specify how the interaction between an individual and the environment produces a process of specific emotions, such as positive, negative, or mixed (Gu, 2016). Based on a qualitative narrative case study on 12 university EFL teachers about how their emotions are shaped in their research life from ecological perspective, Gu (2016) found that these university EFL teachers’ emotions are shaped by their continuous appraisal between the interaction of their research beliefs or goals and ecological systems and suggested adding teachers’ appraisal and strategies of emotional regulation to the person-environmental interaction model. Sun and Li (2014) also analyzed the ecological factors that influence the generation and development theoretically based on the person-environmental interaction model. They explored the concrete role of each ecological system in the development of teacher emotion. The above studies mainly investigated the ecological factors based on Cross and Hong’s (2012) person-environmental interaction model. However, none of them have reached consensus on what the concrete influential factors are in each ecological system. For example, whether teachers’ strategies for regulation of emotions should be included as a factor that influences teacher emotion in the microsystem has not been determined. That is to say, there is a lack of a framework to classify those ecological factors. Ning (2016) divided the influential factors leading to teachers’ positive emotions into internal factors (including professional identity, teaching ideal, and sense of teaching achievement) and external factors (professional title evaluation system and a teaching incentive mechanism). However, this kind of classification is too general and cannot show the presentation of various factors in different ecological systems. Chen (2020) refined the teacher emotion model based on evidence from a review of literature published between 1985 and 2019 using the meta-analysis method. In this model, she concluded three antecedents of teacher emotion, namely, teachers’ personal antecedents (including teachers’ knowledge, values, and skills; teachers’ personality; and teachers’ professional beliefs), contextual antecedents (including sociocultural factors, policy factors, organizational factors, and stakeholder factors), and emotional capacity (emotional labor strategy, emotional intelligence, and emotional regulation). In this model, each factor of different aspects of antecedents is given further explanation, which both show internal-external and personal-environmental reasons for the development of teacher emotion. With this model, how these antecedents shape teacher emotion is shown concretely and comprehensively. Thus, this study will mainly deploy this model, combining Cross and Hong’s (2012) person-environmental interaction model when summarizing the ecological factors that may influence teacher emotional development in the process of participating in a teaching improvement project.

Based on the above literature, it is clear that, recently, an increasing amount of researchers has focused on teacher emotion from an ecological perspective. However, the research object is relatively too focused: most of the researchers took primary and university teachers as participants, while few researchers investigate secondary school English teacher emotion within an ecological system. Liu (2021) advises to expand a research object and focus on primary and secondary school teachers. What is more, most studies on the ecological factors that influence teacher emotional development are not that systematic. The ecological factors elicited in most studies are just scattered in a list and not concluded based on some models or frameworks. Only several studies have presented some models or frameworks, based on which the ecological factors were investigated relatively more systematic. However, existing models and frameworks for analyzing the ecological factors are a little general and broad and not detailed enough. Thus, this study takes Chen’s (2020) teacher emotion model as the basis to investigate and conclude those ecological factors systematically. In addition, the theoretical basis of factors induction is somewhat bias. Furthermore, the depiction of the development of teacher emotion and the investigation of the influential factors are often separated. As for research methods, interview is the data collection method adopted by all qualitative research based on Bronfenbrenner’s ecological system framework, and there are few studies that use multiple methods (e.g., reflective logs, narrative framework) to collect data, reducing the richness of data to a certain extent (Liu, 2021). Therefore, by utilizing the aforementioned five ecological systems and multiple data collection methods, we aim to examine two Chinese senior high school English teachers’ dynamic emotional development during the process of a teaching improvement project, including those factors that may influence their emotions systematically and comprehensively.

## Analytical Framework

**FIGURE 1:**
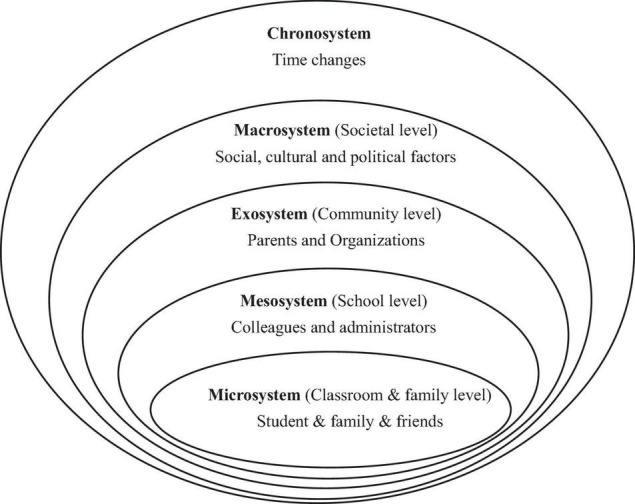
A revised framework adapted from Bronfenbrenner (2005) [as cited in Chen (2020), p. 493].

Considering the complexity of the impacts of social-cultural and environmental factors on the individual emotions, Bronfenbrenner (2005) propounded an ecological systems framework. The surroundings are conceived as nested and complex, consisting of a microsystem, mesosystem, exosystem, macrosystem, and chronosystem (see Figure 1). And this theory “provides a useful theoretical framework for studying the contexts in which teachers exist and develop; thus, it has been used frequently in research on language teacher psychology and practice” (Chu et al., 2021, p. 2). Therefore, the current study adopted this framework to characterize the dynamic emotional development process of two Chinese senior high school English teachers.

The concrete interplay between teacher emotion and each system is as follows:

(1) The microsystem, “which involves the structures and processes taking place in an immediate setting, containing the developing person (e.g., home, classroom, playground)” (Bronfenbrenner, 2005, p. 80). “For teachers as developing persons, they may be influenced by the activities they attend to, the roles that they play, and the relations that they are involved in” (Tao and Jiang, 2021, p. 168). Thus, for teachers in this study, their classroom teaching performance, the roles they play in each lesson, and their relations with students, families, and even with themselves are all involved in this system.
(2) The mesosystem “comprises the linkages and processes taking place between two or more settings containing the developing person” (Bronfenbrenner, 2005, p. 80). It is the connection with the microsystem. For teachers as the developing persons, the mesosystem consists of transactions among students, with other teachers, and with administrators within the school. Other persons who participate actively in the settings also influence their emotions greatly within this system (Tao and Jiang, 2021). Thus, in this study, the transactions of two participants are the essential focus.
(3) The exosystem, “encompasses the linkages and processes taking place between two or more settings, at least one of which does not ordinarily contain the developing person, but in which events occur that influence processes within the immediate setting that does contain that person” (Bronfenbrenner, 2005, p. 80). In short, it refers to the wider social system, such as parent-teacher organizations, government agencies, and other social community. And for the teachers who participate in the teaching improvement project, teacher-expert interaction is the main part of exosystem.
(4) The macrosystem “is defined as an overarching pattern of ideology and organization of the social institutions common to a particular culture or subculture” (Bronfenbrenner, 2005, p. 81). It represents the societal environment in which teachers implement their work according to particular norms, values, regulations, and policies (Cross and Hong, 2012). As for the two teachers in this study, the current teaching material reform with the promulgation of the new high school English curriculum standards in China is the main embodiment of macrosystem.
(5) The chronosystem “permits one to identify the impact of prior life events and experiences, singly or sequentially, on subsequent development” (Bronfenbrenner, 2005, p. 83). It represents changes over time. At different stages of a teaching improvement project, teachers always experience different emotional episodes with different persons in different systems, which affects their emotions.

The reason why the current study chooses the ecological systems framework as analytical framework is that this framework situates a developing person in nested ecological systems and reflects the complex sociocultural world of a teacher and the influences that impact on his or her development (Bronfenbrenner and Morris, 1998). Just as Bronfenbrenner (1979) states, in order to investigate human development, one must take the whole ecological system where growth occurs. In the past 2 decades, with the ecological turn in language teacher education, this framework has been used frequently to resolve the issues on language teacher psychology, such as teacher emotion (Cross and Hong, 2012; Chen, 2019), resilience (Lian, 2015). Existing research has shown that this framework is a useful theoretical framework for studying the contexts where teachers exist and develop. Thus, it is urgently needed in the current study where the research focus is not only teacher emotion but also the contexts where their emotions generated. That is, the ecological framework not only allows researchers and participants themselves to understand their emotion development but also help them gain in-depth insight into the factors influencing their various emotions.

## Methodology

### Research Questions

The research questions to be addressed in the study are:

(1) How did Chinese senior high school English teachers develop their emotions in the interactions with different ecological systems during their participation in a teaching improvement project?
(2) What are the ecological factors that may influence Chinese senior high school English teachers’ emotional development?

### Participants

In accordance with research content, this study used purposeful sampling, that is “selecting information-rich cases to study, cases that, by their nature and substance, will illuminate the inquiry question being investigated” (Patton, 2015, p. 277). More concretely, intensity sampling strategy is used, because it “consists of information-rich cases that manifest the phenomenon of interest intensively (but not extremely)” (p. 293). And “using the logic of intensity sampling, one seeks excellent or rich examples of the phenomenon of interest but not highly unusual cases.” The reason why this study adopts the intensity sampling strategy is that this strategy will enable researchers to obtain the maximum information of teacher emotion development more clearly and carefully, and, based on this, analyze the factors affecting their emotional changes in different ecological systems more deeply. Thus, in the current study, we selected two Chinese senior high school English teachers, Mrs. A and Mrs. B, as research participants. Both participants teach English at one urban key senior high school in Beijing, China. Mrs. A has 26 years of English teaching experience. Mrs. B has 20 years’ experience. In the spring semester of 2021, they participated in an English teaching improvement project carried out cooperatively by their high school, and a key university features teacher education. This project aims to improve teachers’ teaching ability under the background of English education reform to cultivate and develop students’ core competence. The two teachers collaborated to design and teach a 90-min reading and writing lesson. So, inevitably, they would communicate and discuss with each other. In the course of improvement, they participated in an online collective lesson presentation, a trial teaching lesson, and a formal teaching lesson. At each stage, at least two experts from one key university in China observed their lessons and provided comments, guidance, and suggestions. Thus, they maintained close contact with the guidance experts throughout the process. Additionally, one of the experts of the project was also the main researcher of this paper, allowing her to retain an insider’s view. This kind of familiar relationship is convenient for researchers to collect rich and real data (Chen, 2000). The improvement process lasted for a whole semester (4 months), during which the two participants had frequent interactions with people and things in different ecological systems. Their emotions were also displayed differently when facing different emotional episodes. Thus, data on the two English teachers’ emotions collected during the whole process of the teaching improvement project are rich and suitable to deeply analyze their teacher emotion development.

### Research Method

To have a comprehensive exploration of the research questions, this study employed the method of narrative case study. Cases studies are frequently qualitative and interpretative, “generally involve rich contextualization and a deep, inductive analysis of data from a set of participants, sites, or events” (Duff, 2012b, p. 1). They seek depth in their scope and analysis with the goal to particularize and then yield insights of potentially wider relevance and theoretical significance (Duff, 2012a). Thus, case study is appropriate for this study because the methods used can provide a deeper understanding of individual teacher emotion and seek depth in the influential factors. Meanwhile, narrative inquiry “is a way of understanding experience. It is a collaboration between a researcher and participants over time in a place or series of places and in social interaction with milieus” (Clandinin and Connelly, 2000, p. 20). “The focus of narrative inquiry is not only on individuals’ experience but also on the social, cultural, and institutional narratives within which individuals’ experiences are constituted, shaped, expressed, and enacted” (Clandinin and Rosiek, 2007, p. 42). This study focuses on the development of teachers’ individual emotions as well as their interactions with different ecological systems. Therefore, a narrative inquiry is an appropriate method for both inquiring narratively into teacher emotion and the situations where the emotions are generated.

### Data Collection

A narrative framework, interviews, and teachers’ reflective logs were used to collect data in this study.

#### Narrative Framework

Based on Benson’s (2014) description of narrative inquiry frames and Bronfenbrenner’s (2005) five ecological systems, this study designed a narrative framework of the development of teacher emotion. A pilot study was carried out with a teacher from another senior high school who has participated in the improvement project previously. Finally, the different stages and the contents in the framework are as follows (see Table 1).

**TABLE 1:** Stages and contents of the narrative framework of teacher emotion.

Stages	Contents
1. Before the collective lesson presentation	1. Teacher emotion at each stage 2. Key emotional episodes 3. Emotional changes
2. After the collective lesson presentation	
3. Before the trial teaching	
4. After the trial teaching	
5. Before the formal teaching	
6. After the formal teaching	

#### Interviews

Regarding the interview, we conducted two individual structured interviews with each participant. Open-ended interview questions were prepared for each interview to serve as a reference guide for the interviewer. Each interview lasts between 20 and 30 min. The first interview was conducted after the trial teaching, aiming to explore the development of teacher emotion in the period of collective lesson presentation and trial teaching. The second interview was conducted after the formal teaching in order to examine teacher emotion after the whole improvement progress. In order to make sure that the participants have clearer descriptions of their emotions, we provided a list of emotional vocabularies adopted from Parrott (2001), from which they could choose the concrete words. The interviews were audiotaped and transcribed verbatim.

#### Teachers’ Reflective Logs

Additionally, after each period during the teaching improvement project, the teachers were asked to write reflective logs. Totally, the two participants wrote six reflective logs, 6,821 words. Thus, we employed data source triangulation techniques by analyzing these logs to enrich the research findings.

### Data Analysis

All the data were analyzed based on the procedures of content analysis (Barkhuizen and Wette, 2008) and three-step coding (Corbin and Strauss, 2015) in the data analysis with the help of software Nvivo 12 (see Table 2). In opening coding, we attempted to figure out all the sentences related to the participants’ interactions and emotions at different systems. Then, we got 121 nodes (the unit in Nvivo 12). Based on the research questions and Parrott’s (2001) classification of emotions, we categorized the 121 nodes into 68 emotions and initial seven factors (with 12 sub-factors). Finally, in selective coding, we categorized 68 themes into four themes (microsystem, mesosystem, exosystem, and macrosystem) based on Bronfenbrenner’s (2005) ecological system and seven factors into three aspects of antecedents.

**TABLE 2:**
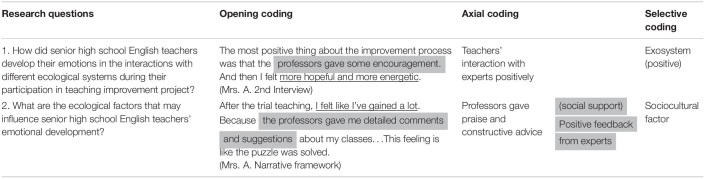
A sample of data coding steps.

*The highlighted parts refer to teachers’ interaction with different ecological systems; the underlined parts refer to teacher emotion developed in corresponding systems.*

In order to guarantee the reliability of this study, the following strategies were adopted:

Firstly, a variety of data collection methods were used in this study to ensure the richness of data to form data triangulation validation. To be more specific, as mentioned above, this study employed data source triangulation by adopting a narrative framework, interviews, and teachers’ reflective logs as data collection methods, which ensure the richness, reliability, and inherent consistency of the data being collected. Secondly, to reduce the influence of cultural politeness on teachers’ expression of their true emotions, all the data were collected by the other researcher, avoiding involving the teacher trainer in the data collection process. And the whole interview process was videotaped. After transcribing the video, the researchers invited the participants to check whether the transcripts were congruent with the emotions they wanted to express as Creswell (2009) has suggested. Thirdly, at the data analysis stage, to guarantee the coding validity and reliability, two researchers independently coded the transcripts and then compared the codes. Whenever there was a disagreement, the two researchers discussed and reconciled the differences. While discussing, we added, deleted, or modified codes and finally reached an agreement. What is more, we invited an “outsider,” who is also a scholar in this field and good at coding data, to analyze the data to make sure that the coding results were consistent.

## Results

Through analysis of the data, the researchers found that the two participants developed 68 emotions, 39 positive and 29 negative emotions. Both two teachers presented their inner emotional change over the time. Mrs. B developed her emotions in twists and turns, from positive to negative and, finally, positive predominantly. While Mrs. A concluded her emotions have evolved from “worry (negative) to confidence (positive).” Below we provide descriptions of teacher emotion generated within different ecological systems, respectively.

### Microsystem

#### Development of Teacher Emotion in Microsystem

The participants reported 20 emotions, consisting of 13 positive (love, expectation, interest, satisfaction, eagerness, anticipation, hope, firmness, open-mindedness, confidence, like, happiness, and progress) and 7 negative emotions (pity, worry, anxiety, tension, impatience, ambivalence, and unsatisfaction) at the microsystem.

The very first emotion both the two teachers expressed was love. Mrs. B explicitly said, “I love teaching and research.” Mrs. A focused on herself more and said, “I like to see my improvement.” This kind of love is one of the reasons why they participated in this project.

The most frequently reported positive emotions are love, expectation, and interest. Among them, the highest frequently reported positive emotion is expectation within this system, especially Mrs. A expressed her expectation to varying degrees; one example is as follows:

*I am very eager to apply the knowledge I have learned, the skills I have learned*, *and the ideas I have learned to my own teaching. I also hope that I can improve higher, improve faster. If my teaching level improved, then students will certainly follow the tide…* (*Mrs. A, 2nd interview*).

From the above transcript, Mrs. A mainly developed her expectation for her teaching directly as well as for students indirectly. And the expression “I am very eager to apply…to my own teaching” also reflects her strong desire for improvement.

The most frequently described negative emotions are pity and worry. And Mrs. B was extremely reflective on her teaching performances. She reported more unpleasant emotions on herself, as can be seen from the following examples,

*…teaching is an art full of pity. I also felt pity for my formal teaching for not performing better…when giving the lesson, I was not only worried that students would not understand well if I do not give enough explanations, but also worried that I gave too detailed and complicated explanations (Mrs. B’s 2nd interview*).

*I felt pity for delaying the first class after the vacations* (*Mrs. B’s narrative framework*).

#### Ecological Factors Influencing Teacher Emotional Development in Microsystem

The development of teacher emotion (both positive and negative) at the microsystem level is closely related to their personal experience and the persons directly related to themselves and their teaching, such as Mrs. A’s father as she mentioned:

*One of the things that influenced my emotion most of the whole process was that my father was ill*, *and I was worried about not being able to give lessons on time (Mrs. A.’ narrative framework).*

Besides, teachers’ positive emotions are mainly derived from their own teaching goals and practice, that is teachers’ professional beliefs of teachers’ personal factors. For example:

*I feel satisfied with the progress I’ve made. I hope to apply what I have learned today to my own classroom and constantly improve my teaching (Mrs. A.’ 2nd interview).*

However, teachers’ negative emotions are mainly related to their unexpected bad teaching performances in the project and their students’ passive and effectiveless learning in class, as is shown in the following example:

*…The student did not read or listen to English at home for a long time during the holiday, so I felt that I had some difficulties in input. Then, I was anxious, because I felt that the student could not understand something in the process of input…I am still a little pity, because I don’t think I was at my best… (Mrs. B.’ 2nd interview)*.

When their own teaching performances were not that good or students’ unacceptable behaviors or struggles with understanding learning contents came out, their negative emotions were elicited.

### Mesosystem

#### Development of Teacher Emotion in Mesosystem

The participants developed 12 emotions within this system. Among them, two (gratefulness and powerfulness) are positive and 10 (anxiety, disappointment, puzzlement, sadness, aggrievement, inactivity, passive, incomprehension, complication, and disgrace) are negative. Gratefulness is the common positive emotion developed by the two participants. Mrs. B developed her gratefulness toward Mrs. A for explaining for her to the school administrators; and Mrs. A not only showed her thankfulness to school leaders, but also, she saw the effect and powerfulness of teachers’ cooperation:

*Mrs. A explained to the school (administrators) that I didn’t adjust class schedule privately. I was grateful that she understood my original intention and explained for me… (Mrs. B.’ 2nd interview)*.

*Anyway, I want to give many thanks to our school’s leaders, who supported our work in the teaching improvement project and, finally, adjusted class schedule for us… Besides, in the whole process, we, two teachers, cooperated a lot; the effect of cooperation is good and powerful… (Mrs. A’ 1st interview)*.

However, more negative emotions were developed than positive emotions in this system. The most frequently mentioned negative emotions are disappointment and anxiety. Relationships and cooperation between the two participants were difficult. Mrs. B expressed her anxiety about the cooperation in her first interview by saying “I felt extremely anxious about cooperation with other teachers because I am not able to do what I really want to do sometimes and I have to make all kinds of adjustments and changes.” When coming to their own individual part of teaching design or giving lessons, they were too self-centered. They seldom gave and took each other’s advice, as Mrs. B complained in her second interview that “though we cooperated to design a class, we still only did our own parts. Even when she (Mrs. A) changed some activities or some designs, she did not tell me or asked for my advice. So, I felt I was in a passive situation and a little disappointed.” Mrs. A also thought that “it was disappointing when there is poor or difficult communication in the cooperation.” So, eventually, they developed more negative emotions than positive emotions in their relationships.

#### Ecological Factors Influencing Teacher Emotional Development in Mesosystem

School climate of organizational factors is the main reason for teacher emotion change in mesosystem. For positive emotions, gratefulness was generated in situations where both Mrs. A and Mrs. B were facing common difficulties and incidents related to their teaching in school, where they got supports from each other or school leaders. They both described an emotional episode where gratefulness was generated. As has been mentioned above, Mrs. A and Mrs. B cooperated to teach this reading and writing lesson in one class. So, they needed to adjust Mrs. B’s class schedule to guarantee that they can have shared free time for the formal teaching. However, teachers are not allowed to adjust class schedule privately in this school. Under the joint efforts of the two teachers and school administrators and related school leaders, they finally adjusted the schedule. According to this episode, it can be seen that the school climate is supportive. And the democratic leadership style of school leaders brings positive effects on teacher emotion. Besides, Mrs. A said, “I felt the power of cooperation” in the narrative framework. This has a high degree of alignment with the outcomes in Cross and Hong (2012) that positive attitudes have a strong relationship with the degree of support provided by colleagues and school administrators.

However, collegial relations could bring mixed emotions for teachers. And when there were differences of opinion or poor communication, more negative emotions emerged, and even occupied a dominant position. Despondently, Mrs. B described how her attempts were denied by Mrs. A in front of her own students.

*I really wanted to do a good job that day (formal teaching) to give students enough input to prepare for the later writing. So I procrastinated a little bit. This is my fault. But before Mrs. A’s lesson, she kept saying with my students that, although last class was extended…That’s it…it just made me feel like…I felt very sad, also a bit aggrieved (Mrs. B.’ 2nd interview).*

In addition, they had different teaching philosophies and styles, also different attitudes toward students. Mrs. B held the opinion of giving students enough language input before output, just as she said above “to give students enough input” and “to prepare for the later writing.” So she thought she had to activate students enough even though procrastinated in a public lesson. However, Mrs. A thought they should present a perfect public lesson, and they could tutor students after class. As she had said in the 2nd interview, “The formal teaching is the display of our progress in the project. Teachers can answer more difficult questions of students after class… Though the reading part was procrastinated, I wanted to ensure my part to continue smoothly and as perfectly as possible.” This kind of comparable degree of investment in the students and in lessons caused the main strained collegial interaction and negative emotions. That is to say, teachers’ professional beliefs of teachers’ personal antecedents have impacts on their collegial relationship as an organizational factor, to some extent, eliciting some negative emotions.

### Exosystem

#### Development of Teacher Emotion in Exosystem

The participants developed the most emotions (28 kinds) in the interaction within this system. Nineteen are positive (gratefulness, hopefulness, confidence, willingness, expectation, adoration, excitement, satisfaction, harvest, luck, optimism, positivity, yearning, determination, happiness, honor, move, encouragement, and perspicuity) and nine (tension, anxiety, depression, shame, pity, tire, aggrievement, discouragement, and champ) are negative emotions. The most frequently mentioned positive emotions are gratefulness, hopefulness, and confidence. These positive emotions are shown in two aspects. On the one hand, they showed extremely positive attitudes toward this teaching improvement project. For example, they both applied actively for this project. They were full of hope and expectation for the promotion this project would bring to them. Mrs. A expressed her gratefulness to this project and the confidence coming along as follows:

*Thanks the collective lesson presentation activity of the teaching improvement project, which changed our understanding of textbooks and teaching methods in group discussions. I felt more confident in my later teaching design of this lesson (Mrs. A.’s 1st reflective log).*

On the other hand, more positive emotions were developed from the interaction between them and the experts. Mrs. A and Mrs. B totally expressed their gratefulness to the experts nine times in different forms, for example:

*I am lucky and grateful to have the chance to communicate with the experts. In the process of discussing with them, I had a better understanding of teaching. I am more confident to implement the core competence to teaching activities (Mrs. A.’s 2nd interview).*

*After the collective lesson presentation, the trial teaching, and formal teaching, my teaching improved a lot. This progress is inseparable from the guidance and help of experts. The guidance and affirmation of the experts make me confident and hopeful about the future design of reading and writing courses. Thanks all the experts (Mrs. B.’s 3rd reflective log).*

The most frequently described negative emotions are tension, anxiety, and depression. They are “nervous” when the experts are listening to their classes. They “felt anxious” that they “may not be able to complete the lesson better in front of the experts.” And Mrs. B even had “a sense of depression when experts didn’t give praise after the trial teaching.”

#### Ecological Factors Influencing Teacher Emotional Development in Exosystem

The development of the participants’ emotions within this system was mainly due to their interaction with experts and the difference of their emotional capacity. Social supports and positive feedback coming from the experts contribute to their positive emotions. Although they inevitably developed negative emotions like tension and anxiety when they were giving lessons in front of the experts, they still described more pleasant feelings after each stage, especially after receiving praise and constructive advice from the experts. Just as Mrs. A said in the interview and the narrative framework:

*The most positive thing about the improvement process was that the professors gave some encouragement. And then I felt more hopeful and more energetic. I have more confidence in myself and my teaching. I think that’s an important aspect of participating in the improvement program… (Mrs. A.’s 2nd interview)*.

*After the trial teaching, I felt like I’ve gained a lot. Because the professors gave me detailed comments and suggestions about my classes…This feeling is like the puzzle was solved (Mrs. A.’s narrative framework).*

The interaction between teachers and experts constitutes the core interaction of the project. The improvement of teachers’ teaching is the core goal of the project. Although they may feel ashamed and pity for not performing better, the above data revealed that the teachers were happy when their efforts had been recognized by the experts. The experts’ advice on their teaching not only brought immediate effects on their feelings but also had a long-term impact on their teaching. Based on what experts had commented on the lessons they gave in the teaching improvement project; the teachers would reflect how to improve their later lessons in future teaching.

However, when they received some negative feedback or they had not fully implemented the experts’ guidance, their emotional capacity would play an important role. Mrs. A’s capacity of emotion regulation is stronger than Mrs. B’s. Although Mrs. A felt a little ashamed that she had not fully implemented the experts’ guidance, she still regulated her emotions immediately and tried to eliminate emotional contradictions and turned the shame into expectation for the next promotion. However, Mrs. B developed more negative emotions like depression, puzzlement, and anxiety. For not fully implementing the experts’ guidance, she also showed unpleasant feelings. But she focused too much on the experience and did not regulate the negative emotion properly.

### Macrosystem

#### Development of Teacher Emotion in Macrosystem

The data showed that, at this level, the teachers developed the least emotions, totally five (interest, eagerness, love, yearning, and gratefulness) positive emotions and three (helplessness, challenge, and difficulty) negative emotions. The teachers developed relatively average positive and negative emotions in this system. They showed positive feelings toward the benefits of using new textbooks, while they felt unpleasant for delaying their teaching planning by some force majors. For example, Mrs. B described an emotional episode where her teaching plans and even teaching performances were influenced by holidays:

*…because of all kinds of reasons that beyond my control, my plans in this project were disrupted. After the Dragon Boat Festival, students had a three-day Gaokao holiday again. Students’ performances were not that ideal. All of these things also made me helpless… (Mrs. A.’s 2nd interview)*.

#### Ecological Factors Influencing Teacher Emotional Development in Macrosystem

In the macrosystem, policy factors are the main factors that influence teachers’ development of emotions. As has been mentioned above, the teachers’ negative feelings were mainly related to the restrictions of social policy, like *Gaokao* and Dragon Boat Festival holiday, both delaying the teachers’ overall planning in the project. What is more, one thing both Mrs. A and Mrs. B showed mixed emotions is the Teaching Material Reform, which is currently one of the important educational reforms. For one thing, they thought that the use of new textbooks would bring some challenges. For another, they love the innovative activity design in new textbooks, which enhances their innovation ability in the process of teaching practice. Mrs. A described her mixed emotional experience at this level:

*…I want to say that the love is to XXX edition new textbook…Now I feel like it’s part of my teaching, even part of my body…This textbook is really, really good. There is a lot of content to dig…But it is very difficult for teachers who use the new edition textbook for the first time. Because using a new teaching material, every day, we need to prepare for lessons seriously. And a lot of relevant information should be sought by ourselves, unlike using old textbooks. So we are pioneers in using this textbook (Mrs. A.’s 2nd interview).*

These findings showed that, even though sometimes, educational reform brings challenges to frontline teachers, they still have shown strong willingness to regulate their emotions to adapt to innovation and regarded this change as an opportunity to improve their teaching.

### Chronosystem

#### Development of Teacher Emotion in Chronosystem

Before the collective lesson presentation, both Mrs. A and Mrs. B were very wishful and really looking forward to this project. But they also felt nervous and puzzled when preparing for the presentation of their teaching plans. This experience was compared as “wade across the stream by feeling the way” (Mrs. B.’s narrative framework). After the presentation, they felt suddenly enlightened and joyful because their “confusion about instructional design was solved” (Mrs. A.’s narrative framework). Meanwhile, Mrs. B also developed negative emotions like chagrin and anxiety for “failing to let the experts better understand the teaching design.”

Before the trial teaching, they still had felt both expectant and nervous. Mrs. B compared this experience as “a novice teacher’s first public lesson.” After the trial teaching, their emotions were not so consistent as they were after the collective lesson presentation. Mrs. A developed more positive emotions than negative emotions at this stage. And most of her positive emotions are related to her interaction with the experts.

Just because of their different emotions developed after the trial teaching, they also had diverse feelings when preparing for the formal teaching. Despite Mrs. A was stressful, she had more confidence than before, while Mrs. B was in a dilemma of adjusting class schedule and revising her teaching design.

After the formal teaching, the two teachers both developed more kinds of positive emotions than at any previous stage. Concretely, they are “much open-minded and hopeful to future teaching,” “happiness, satisfaction, determination, and confidence,” (Mrs. A) “optimism,” and “full of confidence and hope for reading and writing class” (Mrs. B).

**FIGURE 2:**
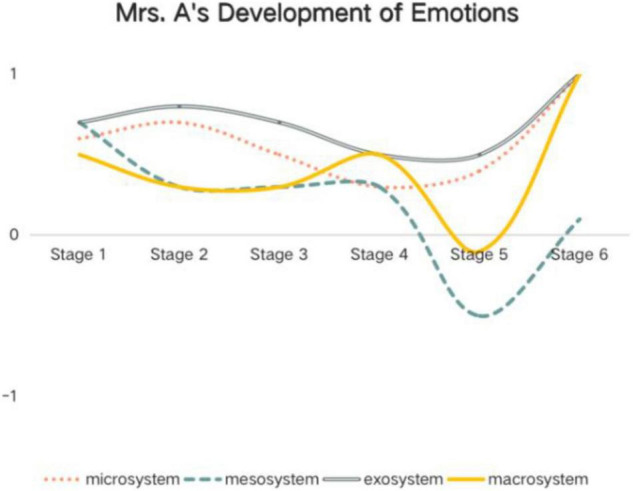
Mrs. A’s development of emotions within different ecological system.

**FIGURE 3:**
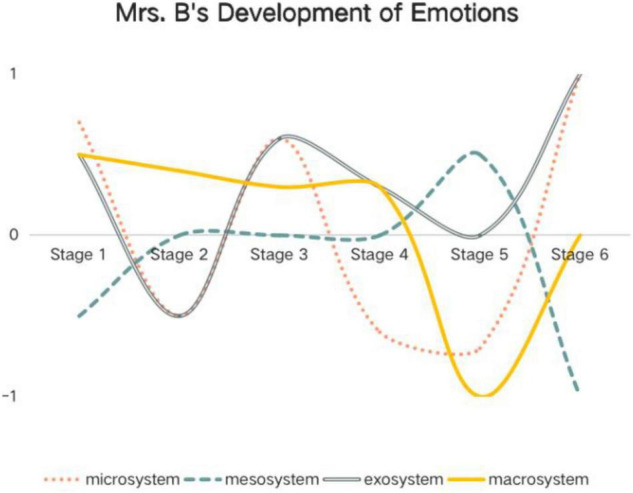
Mrs. B’s development of emotions within different ecological system.

Based on the data above, Mrs. A and Mrs. B’s holistic developments of emotions within different systems at different stages are shown below (Figures 2, 3).

*1 represents totally positive emotion, and*−*1 represents totally negative emotion. The numbers between*−*1 and 1 represent different degrees of positive and negative emotions. Stage 1 = before the collective lesson presentation; Stage 2 = after the collective lesson presentation; Stage 3 = before the trial teaching; Stage 4 = after the trial teaching; Stage 5 = before the formal teaching; Stage 6 = after the formal teaching.*

As is shown, in the whole process, most of Mrs. A’s emotions are positive predominantly. At Stage 4 (after the trial teaching) and Stage 5 (before the formal teaching), her negative emotions developed, stemming from different factors, especially in the interaction with mesosystem and macrosystem. But she regulated her emotions well and quickly. And after the formal teaching, her emotions obviously turned to positive ones.

*1 represents totally positive emotion, and*−*1 represents totally negative emotion. The numbers between*−*1 and 1 represent different degrees of positive and negative emotions. Stage 1 = before the collective lesson presentation; Stage 2 = after the collective lesson presentation; Stage 3 = before the trial teaching; Stage 4 = after the trial teaching; Stage 5 = before the formal teaching; Stage 6 = after the formal teaching.*

Mrs. B’s emotions fluctuated widely. Her positive and negative emotions show symmetrical distribution. And her emotions within different systems also fluctuated to different degrees. Moreover, the final emotions developed in the last stage vary shockingly. Within microsystem and exosystem, she developed extremely positive emotions, while, within mesosystem, extremely negative emotions emerged.

#### Ecological Factors Influencing Teacher Emotional Development in Chronosystem

In the whole process of teaching improvement, different factors influence the participants’ fluctuation of emotions at each stage. Before the collective lesson presentation and the trial teaching, the teachers’ knowledge, values, and skills in these personal factors dominated their emotions and mainly elicited positive emotions like “hope” and “expectation.” During the process of improvement, like after the trial teaching, Mrs. A and Mrs. B’s emotions fluctuated quite differently because of all of the three aspects—personal factors, contextual factors, and emotional capacity. To be more concrete, they are teachers’ professional beliefs, different degrees of experts’ social supports (like experts’ praise and amending advice) and the teachers’ own emotion regulation as mentioned above.

The data revealed that they developed similar pleasant feelings after the formal teaching because of the increase of their knowledge and the improvement of their teaching skills. However, the sources of their negative emotions are quite different. Mrs. B focused more on the formal teaching itself (including her own performance and her colleague’s behavior), like “I felt pity for the class delay,” “sorry for influencing Mrs. A’s lesson,” “very disappointed that Mrs. A told my students that they shouldn’t be influenced by my class delay.” While Mrs. A’s negative emotions are more related to her future teaching. She described in the narrative framework as follows:

*I feel a little ambivalent now…I’m afraid I’ll be stuck in that circle again where I’m not open-minded enough when designing lesson like before…Can I maintain such a state if there is no experts’ guidance and research assistant’s supervision?*

## Discussion

In this study, the teachers mentioned the most emotions at the exosystem level, followed by the microsystem, the mesosystem, and the macrosystem. Teacher emotion in chronosystem is shown through the whole process of the project. Most previous relevant studies found the highest proportion of positive emotions at the nearest microsystem, such as Cross and Hong (2012) and Chen (2017, 2019). In the current study, the teachers’ interaction with the experts at the exosystem comprises the main source of their emotions. And about two thirds are positive emotions. Previous studies investigated overall teacher emotion in a more general environment, such as Chen (2017) explored 53 primary teacher emotions in Hong Kong and Mainland China, and Cross and Hong (2012) investigated two elementary teacher emotions developed in their daily work. However, this study examined teacher emotion in a more concrete context, that is the teaching improvement project. In the project, in addition to teachers’ daily interactions with their students while having class, their interactions with the experts took up a large proportion. At each stage, each teacher communicated 30–60 min with each expert (totally 2). The teachers, indeed, developed pleasant emotions when they got praise and suggestions for promotion. Following the experts’ suggestions, their teaching improved stage by stage, resulting in more positive emotions like confidence and satisfaction at the microsystem. At this point, it is quite consistent with Cross and Hong’s (2012) research that “the systems are nested, so events or structures that exist within one system do influence what happens in another” (p. 964). Past and current exo-events where teachers’ teaching improved have shaped and continue to influence events and emotions in microsystem.

However, it was striking to find that the teachers in this study experienced a large number of negative emotions in mesosystem. In teacher collaboration, team members’ divergent willingness and conceptions of the collaboration space were closely intertwined with teacher emotion (Weddle et al., 2019). And frustration often stems from differing expectations for collaboration and teaching. In the project, at first, Mrs. B designed a complete two-class reading and writing class and wanted to give the two lessons independently. That is to say, she had low willingness of collaborating. She had a passive role during the collaboration. But due to schedule requirements, she had to collaborate with Mrs. A who had strong willingness to collaborate. Besides, Mrs. A felt “responsible for driving the collaborative efforts toward instructional improvement”; she was more positive. Thus, Mrs. B could only design and give one class. So, in this passive cooperative relationship, Mrs. B developed more negative emotions. This kind of negative collegial relationship was also reported by Chen (2019). She argues that, even in harmonious collaboration, teachers still pursue individual excellence. This puts teachers in an emotional dilemma, which is reflected by the negative emotions from the collegial relationship. Regardless of the negative emotions, the two teachers’ positive emotions were generated because of the power of collaboration. They feel powerful regarding collaboration with colleagues and gaining support from leaders. This is consistent with the findings from the study by Erb (2002).

Also, it is worth mentioning that the teachers reported relatively average positive and negative emotions at the macrosystem level, among which three are positive and two are negative. Teaching material reform is the main factor eliciting their negative emotions. It brought difficulties for teaching, such as heavy workload of preparing for new lessons, resulting in the generation of negative emotions. This finding aligns with studies conducted by Yin and Li (2011) that teachers are experiencing high pressure and anxiety during the reforms. Chen and Dai (2014) also mentioned that teachers have to try to change their teaching philosophy and practice to adapt to the reform. The finding is also consistent with the studies by Chen (2016, 2017, 2019). However, most previous studies ignore the positive impact of teaching material reform on teacher emotion. The present study found that the innovative activities in the new textbook provide teachers with a necessary basis and reference for their teaching design. And they love to see that their innovative abilities are enhanced in their use of the new teaching material.

Among all the ecological factors, one point that is important to discuss is that teachers’ different regulating capacities of emotions also influence the development of their emotions, even resulting in diverse holistic evolution of emotions in the whole process. According to Zhang (2009), there is a correlation between the length of teaching years and the emotional regulation of teachers. Teachers with long teaching years have better emotional self-regulation than those with short teaching years. Mrs. A’s teaching years are 6 years longer than Mrs. B’s. Also, this is the second time for Mrs. A to take part in the project. While Mrs. B never participated in this kind of project before. Thus, Mrs. A can regulate and balance her emotions immediately after not-so-perfect performance.

## Conclusion and Implications

The research questions sought to examine two Chinese senior high school English teachers’ dynamic emotional development process in their interaction with different ecological systems during the teaching improvement project and those ecological factors that may influence their emotional development. The analysis reveals the following findings:

Firstly, the two participants developed 68 emotions, 39 positive and 29 negative emotions. And teacher emotion changed with time quite obviously at each stage of the project in the interaction with various systems. They evolved from positive to negative and, finally, positive predominantly. Among all the ecological systems, they developed the most emotions (28 emotions) at exosystem, and they developed the main negative emotions at mesosystem.

Secondly, as for the ecological factors, there are three main factors that may influence teacher emotion, namely personal antecedents, contextual antecedents, and teachers’ emotional capacity. The personal antecedents like teachers’ knowledge, values, and skills and their family conditions and their different professional beliefs are the main factors leading to their change of emotions in microsystem. School climate (collegial relations and leadership style of school) in organizational factors of contextual antecedents is the main reason for teacher emotion development in mesosystem. The development of the participants’ emotions within exosystem was mainly due to the experts’ social supports (e.g., praise and constructive advice from the experts) of contextual antecedents and the difference of teachers’ emotional capacity (teachers’ emotion regulation). In macrosystem, policy factors (e.g., educational reform) of contextual antecedents are the main factors that influence teachers’ development of emotions. And, at different stages in chronosystem, different factors have different roles in the fluctuation of teacher emotion.

This study tells the story of two English teachers who participated in a teaching improvement project and investigates their emotional development in their interaction with ecological systems and those ecological factors that may influence their emotional development in the whole teaching improvement process. By qualitative analysis, this study provides three implications for teacher education.

Firstly, it is necessary for teachers to take part in some teacher training/improving activities. Not only can they adapt to the education reform better, but also gain more confidence in their future teaching by interacting with exosystem.

Secondly, teachers’ interaction with mesosystem is also a crucial part in their emotional development. Furthermore, a teacher education program should not only encourage collegial collaboration but also train teachers how to collaborate positively. Negative collaboration is even counterproductive to their development.

Thirdly, emotional regulation training is also an essential issue. Teachers need to have some strategies of regulating emotions, which, to some extent, ensure teachers’ professional development. Consequently, teacher educators should also focus on the development of teachers’ emotional regulation teaching strategies.

## Limitations and Suggestions

One limitation of this study concerns the generality of the findings. The current study is a case study focusing on two teachers and their emotions. The emotion development of the two participants within different ecological systems can hardly represent the overall senior high school English teacher emotion in each ecological system. The findings can neither reflect most teachers’ attitudes toward the same emotional episodes, such as teacher collaboration. Therefore, the generalization of the results may be limited. It would be interesting for future studies to expand an individual survey to a group survey to gain more universally applicable results. The second suggestion for future studies is to combine some quantitative instruments like a teacher emotion inventory or a questionnaire to promote the generalization of the results.

## Data Availability Statement

The original contributions presented in the study are included in the article/supplementary material, further inquiries can be directed to the corresponding author/s.

## Author Contributions

XS: whole research design and data collection and analysis. LY: data collection and analysis. Both authors contributed to the article and approved the submitted version.

## Conflict of Interest

The authors declare that the research was conducted in the absence of any commercial or financial relationships that could be construed as a potential conflict of interest.

## Publisher’s Note

All claims expressed in this article are solely those of the authors and do not necessarily represent those of their affiliated organizations, or those of the publisher, the editors and the reviewers. Any product that may be evaluated in this article, or claim that may be made by its manufacturer, is not guaranteed or endorsed by the publisher.
